# Supplementary material for: Recombinant cystatin-like protein-based competition ELISA for Trichinella spiralis antibody test in multihost sera
Source: PLoS Negl Trop Dis. 2021 Aug 25;15(8):e0009723. doi: 10.1371/journal.pntd.0009723 (PMC8423253; doi:10.1371/journal.pntd.0009723)
Supplement: S1 Text — Fig A The negative-to-positive ratios of the three MAbs at different dilutions under optimal conditions. The negative-to-positive ratio of 1H9 was higher than those of 6B5 and 7F8 MAbs. The 1H9 was able to compete with the serum that contained antibody against T. spiralis for the available epitopes of rCLP antigens and was suitable for the development of rCLP-cELISA. (DOC) [file pntd.0009723.s005.doc]

**S1 Text**

**Selection of MAbs binding to native epitopes of CLP**

A pooled serum sample from 30 *Trichinella*-free pigs served as the negative control serum, and a pooled serum sample from 3 pigs infected with 50000 larvae of *T. spiralis* (iss 534) at 120 dpi served as the positive control serum. Three MAbs against CLP (1H9, 6B5 and 7F8) were prepared in our previous study [1]. MAbs-biotin were purchased from Sangon Biotech. Co., Ltd. China. The rCLP antigens were prepared as described in our previous study [1]. To determine the ability of MAbs binding to native epitopes of CLP, a competitive ELISA was performed using checkerboard titration to determine the optimum coating concentrations of rCLP, the optimum MAb dilution and the optimum serum dilution. Briefly, 96-well plates were coated with rCLP diluted in carbonate buffer (pH 9.6) and incubated at 4 °C overnight. After three washing steps with phosphate buffered saline supplemented with 0.1% Tween-20 (PBST), test sera (positive control serum or negative control serum) and MAbs were added to the plate in a volume of 100 μL per well and incubated at 37 °C for 1 h. After washing, a 1:500 dilution of HRP-conjugated avidin (Invitrogen, USA) was added and incubated for 30 min at 37 °C. Finally, after three washes, 100 μL of soluble TMB substrate solution (Beijing Solarbio Science & Technology Co., Ltd, China) was added. After 10 min, 0.2 M H_2_SO_4_ solution was added to stop the reaction. The optical density of each well was read at 450 nm (OD_450nm_). The OD_450nm_ ratios of negative control serum to positive control serum (N/P) were calculated. A greater N/P ratio indicated that *T. spiralis* infected sera had a greater ability to block the binding of the MAb to CLP.

The results of the checkerboard titration indicated that the optimum concentrations of the rCLP antigens to 1H9, 6B5 and 7F8 were 1.25 μg/mL, 1.25 μg/mL and 2.50 μg/mL, respectively. In addition, the optimum serum dilution was 1:1. As shown in Fig. S1, when the concentrations of 1H9, 6B5 and 7F8 were 0.30 μg/mL, 0.30 μg/mL and 1.00 μg/mL, respectively, under the conditions of optimum coating concentration and optimum serum dilution, the N/P ratios reached the highest levels. The N/P ratio of 1H9 was higher than those of 6B5 and 7F8, which indicated that 1H9 was able to compete with the serum-containing antibody against *T. spiralis* for the available epitopes of rCLP antigens and was suitable for the development of rCLP-cELISA.

**
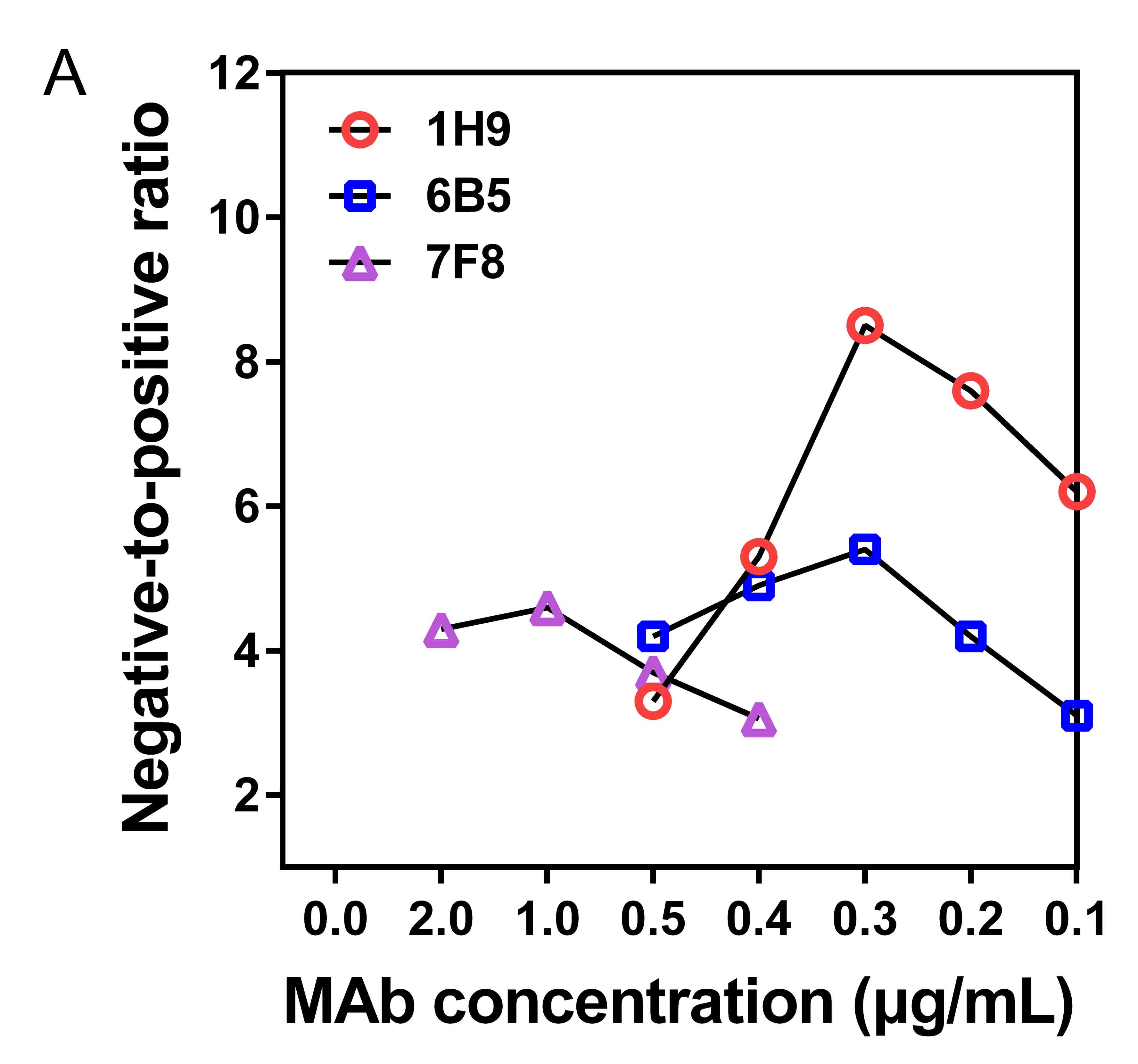
**

**Fig A in S1 Text** **The negative-to-positive ratios of the three MAbs at different dilutions under optimal conditions.**

The negative-to-positive ratio of 1H9 was higher than those of 6B5 and 7F8 MAbs. The 1H9 was able to compete with the serum that contained antibody against *T. spiralis* for the available epitopes of rCLP antigens and was suitable for the development of rCLP-cELISA.

**References**

1. Liu Y, Liu X, Li Y, Xu N, Yang Y, Liu M, et al. Evaluation of a cystatin-like protein of *Trichinella spiralis* for serodiagnosis and identification of immunodominant epitopes using monoclonal antibodies. Vet Parasitol. 2021;297:109127. doi: 10.1016/j.vetpar.2020.109127.
