# Supplementary material for: Recombinant cystatin-like protein-based competition ELISA for Trichinella spiralis antibody test in multihost sera
Source: PLoS Negl Trop Dis. 2021 Aug 25;15(8):e0009723. doi: 10.1371/journal.pntd.0009723 (PMC8423253; doi:10.1371/journal.pntd.0009723)
Supplement: S2 Text — Fig A Diaphragm microscopy for T. murrelli (T5, iss 415) infected mice (100×). The black arrow showed the cyst of T. murrelli (T5, iss 415) larvae. Fig B Genotyping test for two isolates of T. murrelli (iss 415 and iss 35). Lane 1: DL2000 DNA marker; Lane 2: Products for DNA extraction from pooled larvae of T. spiralis (T1, iss 534); Lane 3: Products for DNA extraction from pooled larvae of T. pseudospiralis (T4, iss 141); Lane 4: Products for DNA extraction from pooled larvae of T. murrelli (T5, iss 35); Lane 5: Products for DNA extraction from pooled larvae of T. murrelli (T5, iss 415). (DOC) [file pntd.0009723.s006.doc]

**S2 Text**

**Microscopy and genotyping test in *T. murrelli* (T5, iss 415)**

The diaphragm of infected mouse was taken for compression microscopy at 60 dpi (100×). And we identified *T. murrelli* (T5, iss 415 and iss 35) larvae to the species/genotype level using the multiplex-PCR technique. Primers and procedure were implemented according to methods recommended by the ICT [1].


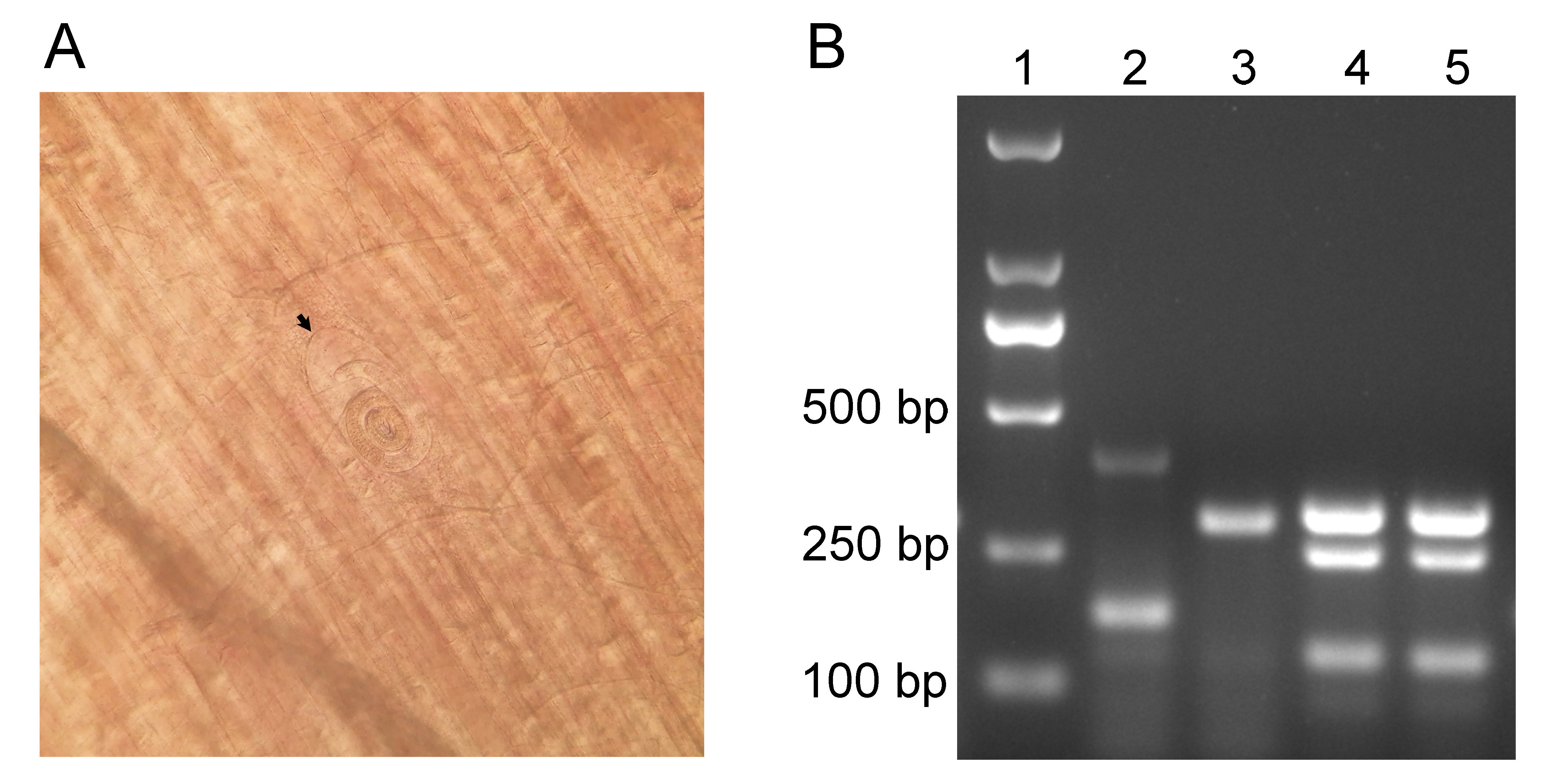
**Fig A in S2 Text** **Diaphragm microscopy for *T. murrelli* (T5, iss 415) infected mice (100×).** The black arrow showed the cyst of *T. murrelli* (T5, iss 415) larvae.

**Fig B in S2 Text** **Genotyping test for two isolates of *T. murrelli* (iss 415 and iss 35).** Lane 1: DL2000 DNA marker; Lane 2: Products for DNA extraction from pooled larvae of *T. spiralis* (T1, iss 534) ; Lane 3: Products for DNA extraction from pooled larvae of *T. pseudospiralis* (T4, iss 141) ; Lane 4: Products for DNA extraction from pooled larvae of *T. murrelli* (T5, iss 35) ; Lane 5: Products for DNA extraction from pooled larvae of *T. murrelli* (T5, iss 415).

**References**

1. Pozio E, Zarlenga D. International Commission on Trichinellosis: Recommendations for genotyping *Trichinella* muscle stage larvae. Food Waterborne Parasitol. 2019;15:e00033. doi: 10.1016/j.fawpar.2018.e00033.
