# Supplementary material for: Recombinant cystatin-like protein-based competition ELISA for Trichinella spiralis antibody test in multihost sera
Source: PLoS Negl Trop Dis. 2021 Aug 25;15(8):e0009723. doi: 10.1371/journal.pntd.0009723 (PMC8423253; doi:10.1371/journal.pntd.0009723)
Supplement: S1 Table — (DOC) [file pntd.0009723.s007.doc]

**S1 Table**

**Comparison of rCLP-cELISA with ES-iELISA and artificial digestion method in experimentally infected swine**

| Doses ^a^ | rCLP-cELISA | | | ES-iELISA | | Artificial digestion | Larvae burden |
| --- | --- | --- | --- | --- | --- | --- | --- |
|  | PI ratio  (%) | *T. spiralis* Ab Status | | S/P value ^d^ | *T. spiralis* Ab Status | Larvae burden in diaphragm ^e^ (larvae/100 g tissue) | The mean LPG in the group ^f^ |
| 50 | 69.90 ± 1.49 | | + ^b^ | 1.95 ± 0.06 | + ^b^ | 2.00 ± 0.00 | 0.005 ± 0.00 |
|  | 67.61 ± 2.35 | | + | 1.71 ± 0.04 | + | 0.00 ± 0.00 |  |
|  | 18.03 ± 1.97 | | - ^c^ | 0.21 ± 0.03 | - ^c^ | 0.00 ± 0.00 |  |
|  | 56.83 ± 1.32 | | + | 0.58 ± 0.11 | + | 0.00 ± 0.00 |  |
|  | 39.60 ± 3.45 | | - | 0.63 ± 0.04 | + | 0.00 ± 0.00 |  |
|  | 23.90 ± 2.60 | | - | 0.18 ± 0.05 | - | 0.00 ± 0.00 |  |
| 100 | 60.40 ± 2.63 | | + | 1.10 ± 0.10 | + | 1.00 ± 0.00 | 0.025 ± 0.00 |
|  | 62.58 ± 2.45 | | + | 1.03 ± 0.10 | + | 2.00 ± 0.00 |  |
|  | 65.71 ± 2.06 | | + | 0.99 ± 0.04 | + | 3.00 ± 0.00 |  |
|  | 48.35 ± 1.63 | | - | 1.14 ± 0.06 | + | 0.00 ± 0.00 |  |
|  | 70.02 ± 1.58 | | + | 1.74 ± 0.05 | + | 8.00 ± 0.00 |  |
|  | 71.11 ± 3.04 | | + | 2.02 ± 0.09 | + | 1.00 ± 0.00 |  |
| 200 | 67.52 ± 2.34 | | + | 1.70 ± 0.02 | + | 1.00 ± 0.00 | 0.423 ± 0.05 |
|  | 64.55 ± 1.63 | | + | 1.72 ± 0.03 | + | 0.00 ± 0.00 |  |
|  | 63.42 ± 3.08 | | + | 1.63 ± 0.02 | + | 0.00 ± 0.00 |  |
|  | 60.86 ± 2.09 | | + | 1.73 ± 0.03 | + | 2.00 ± 0.00 |  |
|  | 60.00 ± 2.84 | | + | 1.44 ± 0.04 | + | 236.00 ± 7.77 |  |
|  | 71.46 ± 2.50 | | + | 1.72 ± 0.04 | + | 0.00 ± 0.00 |  |

Results are mean ± SD (n = 3)

^a^ Doses: larval inoculation dose in swine.

^b^ The *T. spiralis* antibody test is positive.

^c^ The *T. spiralis* antibody test is negative.

^d^ The S/P value and threshold were calculated according to the manufacturer’s instructions (Qiagen, Germany).

^e^ Larvae per 100 g of diaphragm tissues from infected swine.

^f^ The larvae per gram of diaphragm tissues from sera-diagnosis positive swine in ES-iELISA (Qiagen, Germany).
