# Supplementary material for: Recombinant cystatin-like protein-based competition ELISA for Trichinella spiralis antibody test in multihost sera
Source: PLoS Negl Trop Dis. 2021 Aug 25;15(8):e0009723. doi: 10.1371/journal.pntd.0009723 (PMC8423253; doi:10.1371/journal.pntd.0009723)
Supplement: S2 Table — (DOC) [file pntd.0009723.s008.doc]

**S2 Table**

**Performance of ES-iELISA for detecting experimentally infected swine induced by various doses of *T. spiralis***

| Infection dose (ML ^a^) | Mean infection intensity (LPG ^b^) | Serocoversion (dpi ^c^) | Reference |
| --- | --- | --- | --- |
| 50 | 0.87 – 233.00 ^d^ | 42 - 56 | [1] |
| 50 | 0.005 ^d^ | 35 | [2] |
| 50 | 0.02 ^e^ | 45 | [3] |
| 100 | 0.00 – 0.01 ^f^ | 79 - 86 | [4] |
| 100 | 1.62 – 6.50 ^g^ | 35 - 49 | [5] |
| 100 | 0.03 ^e^ | 45 | [3] |
| 200 | 3.00 ^h^ | 40 | [6] |
| 200 | 0.44 ^e^ | 35 | [3] |
| 200 | - ^i^ | 35 - 90 | This study ^*^ |
| 400 | 1.34 ^e^ | 35 | [3] |
| 400 | - ^i^ | 30 - 45 | This study |
| 500 | 11.07 – 303.33 ^d^ | 35 - 49 | [1] |
| 500 | 0.0075 – 0.7575 ^f^ | 65 - 72 | [4] |
| 600 | 68.00 ^e^ | 28 | [7] |
| 600 | 4.27 ^e^ | 35 | [3] |
| 600 | - ^i^ | 30 | This study |
| 800 | 5.08 ^e^ | 30 | [3] |

^a^ ML: muscle larvae.

^b^ LPG: larvae per gram of muscle tissue.

^c^ dpi: days post infection.

^d^ diaphragm and tongue (mixed together).

^e^ diaphragm.

^f^ diaphragm, tongue, masseter, intercostals, psoas and rectus abdominis (mixed together).

^g^ tongue.

^h^ tongue, diaphragm, masseter, shoulder, foreleg, abdomen, hind leg, intercostal and filet.

^i^ -: no test.

^*^ The ES-iELISA used in this study was a commercial ELISA kit.

**References**

1. van der Leek ML, Dame JB, Adams CL, Gillis KD, Littell RC. Evaluation of an enzyme-linked immunosorbent assay for diagnosis of trichinellosis in swine. Am J Vet Res. 1992;53(6):877.

2. Forbes LB, Appleyard GD, Gajadhar AA. Comparison of synthetic tyvelose antigen with excretory-secretory antigen for the detection of trichinellosis in swine using enzyme-linked immunosorbent assay. J Parasitol. 2004;90(4):835-840. doi: 10.1645/GE-187R.

3. Wang N, Bai X, Ding J, Lin J, Zhu H, Luo X, et al. *Trichinella* infectivity and antibody response in experimentally infected pigs. Vet Parasitol. 2020:109111-109111. doi: 10.1016/j.vetpar.2020.109111.

4. Smith HJ. Evaluation of the ELISA for the serological diagnosis of trichinosis in Canadian swine. Can J Vet Res.. 1987;51(2):194-7.

5. Gamble HR. Detection of trichinellosis in pigs by artificial digestion and enzyme immunoassay. J Food Prot. 1996;59(3):295-8. doi: 10.4315/0362-028X-59.3.295.

6. Nockler K, Serrano FJ, Boireau P, Kapel C, Pozio E. Experimental studies in pigs on *Trichinella* detection in different diagnostic matrices. Vet Parasitol. 2005;132(1-2):85-90. doi: 10.1016/j.vetpar.2005.05.033.

7. Reiterova K, Dubinsky P, Klimenko VV, Tomasovicova O, Dvoroznakova E. Comparison of *Trichinella spiralis* larva antigens for the detection of specific antibodies in pigs. Vet Med-Czech. 1999;44(1):1-5.
