# Supplementary material for: Recombinant cystatin-like protein-based competition ELISA for Trichinella spiralis antibody test in multihost sera
Source: PLoS Negl Trop Dis. 2021 Aug 25;15(8):e0009723. doi: 10.1371/journal.pntd.0009723 (PMC8423253; doi:10.1371/journal.pntd.0009723)
Supplement: S3 Table — (DOC) [file pntd.0009723.s009.doc]

**S3 Table**

**Larvae burden in diaphragm of *T. spiralis* (iss 534) infected swine using artificial digestion**

| Doses ^a^ | LPG ^b^ | Ref. | Doses ^a^ | LPG ^b^ | Ref. |
| --- | --- | --- | --- | --- | --- |
| 10,000 | 420.86 ± 75.24 | [1] ^c^ | 400 | 1.34 ± 0.77 | [1] |
| 1000 | 46.70 ± 8.41 | [1] | 200 | 0.423 ± 0.05 | This study ^d^ |
| 800 | 5.08 ± 2.33 | [1] | 100 | 0.025 ± 0.00 | This study |
| 600 | 4.27 ± 2.10 | [1] | 50 | 0.005 ± 0.00 | This study |

^a^ Doses: larval inoculation dose in swine.

^b^ Larvae per 100 g of diaphragm tissues from infected swine.

^c^ The isolate of *T. spiralis* (iss 534), the specie of pig, the experimental method of artificial infection and the diaphragm tissues sampling date (120 days post infection) were consistent with our study.

^d^ The data was shown in S1 Table in the supplementary material.

**References**

1. Wang N, Bai X, Ding J, Lin J, Zhu H, Luo X, et al. *Trichinella* infectivity and antibody response in experimentally infected pigs. Vet Parasitol. 2020:109111-109111. doi: 10.1016/j.vetpar.2020.109111.
