# Supplementary material for: Recombinant cystatin-like protein-based competition ELISA for Trichinella spiralis antibody test in multihost sera
Source: PLoS Negl Trop Dis. 2021 Aug 25;15(8):e0009723. doi: 10.1371/journal.pntd.0009723 (PMC8423253; doi:10.1371/journal.pntd.0009723)
Supplement: S4 Table — (DOC) [file pntd.0009723.s010.doc]

**S4 Table**

**Prevalence of *T. spiralis* in rats trapped on pig farms with active transmission of *T. spiralis***

| Total number of pig farms | Percentage of infected rats (%) | Mean intensity of invasion (LPG ^a^) | Reference |
| --- | --- | --- | --- |
| 3 | 7.69 – 30.36 | 1.78 – 4.25 | [1] |
| 2 | - ^b^ | - ^b^ |
| 6 | 2.00 | NR ^c^ | [2] |
| 1 | - ^b^ | - ^b^ |
| 1 | 42.40 | 293.20 | [3] |

^a^ LPG: larvae per gram of muscle tissue.

^b^ -: not detected.

^c^ NR: not reported.

**References**

1. Bilska-Zajac E, Rozycki M, Antolak E, Belcik A, Gradziel-Krukowska K, Karamon J, et al. Occurrence of *Trichinella* spp. in rats on pig farms. Ann Agr Env Med. 2018;25(4):698-700. doi: 10.26444/aaem/99555.

2. Stojcevic D, Zivicnjak T, Marinculic A, Marucci G, Andelko G, Brstilo M, et al. The Epidemiological Investigation of *Trichinella* Infection in Brown Rats (Rattus norvegicus) and Domestic Pigs in Croatia Suggests That Rats are not a Reservoir at the Farm Level. J Parasitol. 2004;90(3):666-670. doi: 10.1645/GE-158R.

3. Leiby DA, Duffy CH, Murrell KD, Schad GA. *Trichinella spiralis* in an agricultural ecosystem: transmission in the rat population. J Parasitol. 1990;76(3):360-4. doi: 10.2307/3282667.
