# Supplementary material for: Recombinant cystatin-like protein-based competition ELISA for Trichinella spiralis antibody test in multihost sera
Source: PLoS Negl Trop Dis. 2021 Aug 25;15(8):e0009723. doi: 10.1371/journal.pntd.0009723 (PMC8423253; doi:10.1371/journal.pntd.0009723)
Supplement: Attachment — Submitted filename: Revision Note.doc [file pntd.0009723.s011.doc]

**Dear Co-Editor in Chief of PLOS Neglected Tropical Diseases**

On behalf of my co-authors, we thank you very much for giving us an opportunity to revise our manuscript. We appreciate reviewers’ comments on our manuscript entitled “Recombinant cystatin-like protein-based competition ELISA for *Trichinella spiralis* antibody test in multihost sera” (PNTD-D-21-00821). We studied the reviewers’ comments and suggestions carefully, and revised the manuscript and answered point-by-point below. All changes are highlighted in the revised manuscript, covered by yellow. We hereby resubmit the manuscript for your kind consideration of publication, and hope it is acceptable for publication in the journal.

**Reviewer #1**

The Msc deals with the comparison of the ES-iELISA (actual gold standard) and the newly in house developed rCLP-cELISA for the detection of Ab against *Trichinella*. This research is important in the field and better diagnostic tools for *Trichinella* detection are needed.

**Response:** Thanks very much for reviewers’ comments.

**Comment 1:** Why authors choose to focus only on the specificity by infected pork with only three low doses?

**Response:** Thank you very much for your question.

Serum samples (n = 920) collected from backyard pigs from Hubei province of China were used to assess the sensitivity and specificity of the rCLP-cELISA in the field. The agreement between rCLP-cELISA and ES-iELISA in field swine sera was assessed by Cohen’s κ analysis (**Table 2, in the manuscript**). A substantial agreement (κ = 0.7963) was observed between the two methods. In **Table 3 of the manuscript**, field samples were classified as true positive or negative according to their larvae burden in diaphragm. The results showed that the proposed rCLP-cELISA are high sensitivity (se: 1.0000) with specificity of 0.9956.

On the other hand, antibody kinetics of *T. spiralis* infection in swine at various stages were determined using serum samples that obtained from three different infection doses (200, 400 and 600 larvae per pig). Based on the following three important considerations, we focus the performance of the rCLP-cELISA on these three dose groups.

Fig R1. Kinetics of signal-to-positive (S/P) values in ELISA employing rCLP (A, C) or ES antigens (B, D) of serum samples from pigs experimentally infected with 50,000 larvae (A, B) or 1000 larvae (C, D) of *Trichinella spiralis*. It was cited from **our previous study**: Liu Y, Liu X, Li Y, et al. Evaluation of a cystatin-like protein of *Trichinella spiralis* for serodiagnosis and identification of immunodominant epitopes using monoclonal antibodies, Vet Parasitol. 2020:109127. http://doi.org/10.1016/j.vetpar.2020.109127

**1.** In our previous study ^[1]^, we developed an indirect ELISA employing rCLP (rCLP-iELISA), which was preliminarily validated using infected swine sera compared with ES-iELISA (Qiagen). The specific IgG can be detected at 17 dpi for 50,000 larvae group (**Fig R1A**) or at 19 dpi for 1000 larvae group (**Fig R1C**). For comparison, ES-iELISA can detect the IgG at 21 dpi for 50,000 larvae group (**Fig R1B**) or at 25 dpi for 1000 larvae group (**Fig R1D**). Besides, serological positivity of rCLP-iELISA in all of the groups lasted up to 120 dpi (**Fig R1A and R1C**), which was similar to that of ES-iELISA. Above all, the serological antibody detection method based on rCLP has a good performance for infected pig serum samples at high dose (≥ 1000 larvae per pig) of infection.

S3 Table Larvae burden in diaphragm of *T. spiralis* (iss 534) infected swine using artificial digestion

| Doses ^a^ | LPG ^b^ | Ref. | Doses ^a^ | LPG ^b^ | Ref. |
| --- | --- | --- | --- | --- | --- |
| 10,000 | 420.86 ± 75.24 | [2] ^c^ | 400 | 1.34 ± 0.77 | [2] |
| 1000 | 46.70 ± 8.41 | [2] | 200 | 0.423 ± 0.05 | This study ^d^ |
| 800 | 5.08 ± 2.33 | [2] | 100 | 0.025 ± 0.00 | This study |
| 600 | 4.27 ± 2.10 | [2] | 50 | 0.005 ± 0.00 | This study |

^a^ Doses: larval inoculation dose in swine.

^b^ Larvae per 100 g of diaphragm tissues from infected swine.

^c^ The isolate of *T. spiralis* (iss 534), the specie of pig, the experimental method of artificial infection and the diaphragm tissues sampling date (120 days post infection) were consistent with our study. It was cited from **previous study of our laboratory**: Wang N, Bai X, Ding J, Lin J, Zhu H, Luo X, et al. *Trichinella* infectivity and antibody response in experimentally infected pigs. Vet Parasitol. 2020:109111. http://doi.org/10.1016/j.vetpar.2020.109111

^d^ The data was shown in **S1 Table** in the supplementary material.

Fig R2. Kinetics of PI ratios in rCLP-cELISA (A, B, C) and the percentages of swine (D) that scored positive or negative as determined by artificial digestion in low-dose groups. It was cited from **the manuscript of Figs 1D, 1E, 1F and 2A**.

**2.** Artificial digestion test is mandatory for *T. spiralis* larvae test when pork products are put into markets, the limit of detection (LOD) of which was 1.0 LPG ^[3]^. Besides, pigs harbouring as few as 1 larva/100 g of tissue (0.01 LPG) have been detected by ES-iELISA ^[3]^. When the dose of infection was above 1000 larvae per pig, the LPG was more than 46.70 (**S3 Table**) that was much higher than the LOD of artificial digestion and than that of ES-iELISA. Our previous study showed that, the serological antibody detection method based on rCLP had a good performance for infected swine sera of the group higher than this dose (**Fig R1**). Thus, in this study, we focused on the performance of the proposed rCLP-cELISA in low-dose (< 800 larvae per pig) infected swine sera. According to **S3 Table**, we showed antibody curves (**Fig R2**) at 3 dose groups of artificial infection, including 600 larvae (LPG was 4.27 ± 2.10), 400 larvae (LPG was 1.34 ± 0.77) and 200 larvae (LPG was 0.423 ± 0.05) groups. The larvae burden in diagram tissues of 400-dose group was close to the LOD of artificial digestion ^[3]^. It was worth noting that of the 6 infected swine in 200-dose group, only 3/6 pigs were confirmed to be *T. spiralis* larvae test positive in diagram tissues (**Fig R2D**), thus antibody test results of these 3 pigs were showed in the antibody curve test (**Fig R2C**).

Fig R3. Kinetics of S/P values in ES-iELISA (A) or PI ratios in rCLP-cELISA (B) and the percentages of swine (C) that scored positive or negative as determined by artificial digestion in low-dose groups. It was cited from **the manuscript of Figs1C, 1F and 2A**.

**3.** As shown in **Fig R3**, antibody curves of *T. spiralis* larvae test positive pigs in 200-dose group were tested by rCLP-cELISA as well as ES-iELISA. All three pigs were serologically positive at 90 dpi using rCLP-cELISA and ES-iELISA, which was close to our last sampling point (120 dpi). In fact, we also tried to plot antibody curves of the 5 *T. spiralis* larvae test positive pigs in 100-dose group (LPG was 0.025 ± 0.00, **in S3 Table**), and the larvae burden in diagram tissues of 100-dose group was close to the LOD of ES-iELISA (0.01 LPG) ^[3]^. However, the results showed that all these pigs were serologically positive at 120 dpi, no matter using rCLP-cELISA or ES-iELISA.

Therefore, in the manuscript, we choose to focus on the analytical performance of the rCLP-cELISA in antibody curves in three doses of infected swine (600, 400, and 200 larvae per pig), as well as in the accuracy test in three low doses of infected swine (200, 100, and 50 larvae per pig).

The description of the results of field serum samples is provided in line 251-256 as well as in Tables 2 and 3. The consideration for the choice of infective doses for antibody curves is provided in line 341-343 and line 347-352. The new S3 Table is provided as supplemented material, and corresponding description is provided in line 698-699. All relevant descriptions are provided in the revised manuscript, highlighted by yellow.

**References**

(1) Liu Y, Liu X, Li Y, et al. Evaluation of a cystatin-like protein of *Trichinella spiralis* for serodiagnosis and identification of immunodominant epitopes using monoclonal antibodies, Vet Parasitol. 2020:109127. http://doi.org/10.1016/j.vetpar.2020.109127

(2) Wang N, Bai X, Ding J, Lin J, Zhu H, Luo X, et al. *Trichinella* infectivity and antibody response in experimentally infected pigs. Vet Parasitol. 2020:109111. http://doi.org/10.1016/j.vetpar.2020.109111

(3) OIE Terrestrial Manual, [Trichinellosis (infection with *Trichinella* spp.)](https://www.oie.int/fileadmin/Home/eng/Health_standards/tahm/3.01.20_TRICHINELLOSIS.pdf), 2019. Chapter 3.1.20, Available from : https://www.oie.int/standard-setting/terrestrial-manual/access-online/

**Comment 2:** Figure 3:I suggest to change for curves, the differents dot are not easy to read.

**Response:** Thank you very much for your suggestion. The new **Fig 3** is changed for curves, and we present the four dose groups in two graphs including **Fig 3A** and **3B**. The relevant descriptions are provided in line 274-277 and line 616-618 of the revised manuscript, highlighted by yellow.

Fig 3 Antibody kinetics of *Trichinella spiralis* infection in mice determined by rCLP-cELISA.

The cut-off value represented by the dotted line was evaluated on the basis of PI ratios (S3 Fig). Kinetics of PI ratios in the rCLP-cELISA of serum samples from mice experimentally infected with 10 larvae (A), 20 larvae (A), 200 larvae (B) or 400 larvae (B) of *Trichinella spiralis*.

**Comment 3:** Figure 3: To me, on this figure, the detection for the 200 larvae group is ok at J17 but not at J21. So I think the detection is at J30. But do authors have data between J21 and J30? If no, according to these results, we can consider the detection at J30.

**Response:** Thank you very much for your suggestion. We re-examine the original data and find that 1/3 of the mice in the 17 dpi group is serological positive for antibodies, as is that in the 21 dpi group. Considering that we did not design sampling points between 21 and 30 dpi, in **Fig 3B**, PI ratios exceed the threshold at 14 dpi followed by a decrease, and again exceed the cut-off value at **30 dpi** (200 larvae). The relevant descriptions are provided in line 274-277 and line 616-618, highlighted by yellow.

**Comment 4:** Figure 4: Legends of A and B at the same time is not easy

**Response:** Thank you very much for your suggestion. The legend of **Fig 4** has been redrafted as “Preliminary validation of the rCLP-cELISA in mice infected with different species/genotypes of *Trichinella*. A. PI ratios of serum samples of mice infected with 17 isolates of *Trichinella*. The cut-off value represented by the dotted line. The group value was the mean ± SD of three independent experiments (n = 3). B. Analyses of western blot using crude worm extract antigens of 17 isolates of *Trichinella*.” in line 619-624 of the revised manuscript, highlighted by yellow.

**Comment 5:** Figure 4: What are the rounds? Error bars cannot be seen.

**Response:** Thank you very much for your suggestion. **Fig 4A** is reprinted as a color image to show the error bar more clearly. The group value was the mean ± SD of three independent experiments (n = 3).

Fig 4 Preliminary validation of the rCLP-cELISA in mice infected with different species/genotypes of *Trichinella*. A. PI ratios of serum samples of mice infected with 17 isolates of *Trichinella*. The cut-off value represented by the dotted line. The group value was the mean ± SD of three independent experiments (n = 3). B. Analyses of western blot using crude worm extract antigens of 17 isolates of *Trichinella*.

**Comment 6:** Discussion: First paragraph is introduction and should be deleted

**Response:** Thank you very much for your suggestion. The first paragraph of discussion in the original manuscript has been deleted.

**Comment 7:** Discussion: Lines 370-371: To me decreasing from 600 to 200 is not a strong reduction in porks. So it is logical to have the same resuIts.

**Response:** Thank you very much for your question. According to **S3 Table**, when the infective dose was reduced from 600 to 200 larvae per pig, the LPG of diaphragm tissues was reduced from 4.27 ± 2.10 to 0.423 ± 0.05. With the load of larvae in muscles reduced 10-fold, seroconversion for continuous serological positivity was delayed from 35 to 90 dpi in rCLP-cELISA and from 30 to 90 dpi in ES-iELISA (**Fig 1, in the manuscript**). In other words, continuous serological positivity was delayed by **60** days using ES-iELISA and by **55** days using rCLP-cELISA. As shown in **Comment 1**, The specific IgG can be detected at 17 dpi for 50,000 larvae group or at 19 dpi for 1000 larvae group, serological positivity was delayed by only 2 days. Similar results (a 4-day delay) were also detected in ES-iELISA (**Fig R1**). Besides, the 5 *T. spiralis* larvae test positive pigs in 100-dose group (LPG was 0.025 ± 0.00, **in S3 Table**) were serologically positive at last sampling time point (120 dpi), no matter using rCLP-cELISA or ES-iELISA. Therefore, we presented serological test results from three low-dose groups (600, 400 and 200 larvae per pig), and the phenomenon of a delay in seroconversion in swine was consistent with previous studies ^[1-3]^. The relevant descriptions are provided in line 235-238 and line 358-362, highlighted by yellow.

**References**

(1) Bruschi F, Gómez-Morales MA, Hill DE. International Commission on Trichinellosis: Recommendations on the use of serological tests for the detection of *Trichinella* infection in animals and humans. Food Waterborne Parasitol. 2019;14:e32. http://dx.doi.org/10.1016/j.fawpar.2018.e00032

(2) Nockler, K., Reckinger, S., Broglia, A., Mayer-Scholl, A., & Bahn, P. Evaluation of a Western Blot and ELISA for the detection of anti-*Trichinella*-IgG in pig sera. Vet Parasitol,2009; 163(4), 341-347. http://dx.doi.org/10.1016/j.vetpar.2009.04.034

(3) Wang N, Bai X, Ding J, Lin J, Zhu H, Luo X, et al. *Trichinella* infectivity and antibody response in experimentally infected pigs. Vet Parasitol. 2020:109111. http://doi.org/10.1016/j.vetpar.2020.109111

Special thanks to the reviewer for your helpful comments.

**Reviewer #2**

The manuscript titled" Recombinant cystatin-like protein-based competition ELISA for *Trichinella spiralis* antibody test in multihost sera" by Liu and his colleagues developed a novel and sensitive cELISA for the detection of *Trichinella spiralis* infection. The research is of interest for the relative studies.

**Response:** Thanks very much for reviewers’ comments.

**Comment 1:** There are some grammatical and spelling errors in the manuscript. For example, in line 33, "Conclusios" should be "Conclusions"; in lines 165 "2.0 M H2S04" should be "0.2 M H2S04"; in line 414" clonorchis sinensis" should be "Clonorchis sinensis".

**Response:** Thank you very much for your suggestion. "Conclusios" has been changed to "Conclusions" in line 33. "2.0 M H_2_S0_4_" has been changed to "0.2 M H_2_S0_4_" in line 167. "*clonorchis sinensis*" has been changed to "*Clonorchis sinensis*" in line 404, line 409, line 411 and line 415. The relevant descriptions are provided in the revised manuscript, highlighted by yellow. English-writing has been checked carefully.

Special thanks to the reviewer for your helpful comments.
